# Supplementation of Ascorbic Acid in Weanling Horses Following Prolonged Transportation

**DOI:** 10.3390/ani2020184

**Published:** 2012-04-16

**Authors:** Sarah Ralston, Michelle Stives

**Affiliations:** Department of Animal Sciences, School of Environmental and Biological Sciences, Rutgers, The State University of New Jersey, 84 Lipman Drive, New Brunswick, NJ 08901, USA; E-Mail: michelle.stives.dvm@gmail.com

**Keywords:** ascorbic acid, vaccine response, transportation stress

## Abstract

**Simple Summary:**

Horses normally synthesize adequate amounts of ascorbic acid (vitamin C) in their liver to meet their needs for the vitamin. However, prolonged stress results in low plasma concentrations and reduced immune function. Weanling horses were supplemented with ascorbic acid for 5 or 10 days or no ascorbic acid (4 per group) following 50+ hours of transportation. Supplementation caused increases in plasma concentrations but both supplemented groups had decreased plasma ascorbic acid for 1 to 3 weeks following cessation of supplementation, possibly due to suppressed synthesis. Supplementation of ascorbic acid following prolonged stress will increase plasma concentrations, but prolonged supplementation should be avoided.

**Abstract:**

Though horses synthesize ascorbic acid in their liver in amounts that meet their needs under normal circumstances, prolonged stress results in low plasma concentrations due to enhanced utilization and renal excretion and can reduce immune function. It was hypothesized that plasma ascorbic acid could be maintained in weanling horses by oral supplementation following prolonged transportation. Weanlings were supplemented with no ascorbic acid (Tx 0: n = 4), 5 grams ascorbic acid twice daily for 5 days (Tx 1: n = 4) or for 10 days (Tx 2: n = 4) following >50 hours of transportation. Supplementation caused slight (*P* < 0.2) increases in plasma ascorbic acid concentrations. Both supplemented groups had decreased (*P* < 0.05) plasma concentrations for 1 to 3 weeks following cessation of supplementation, possibly due to increased renal excretion or suppressed hepatic synthesis. Supplementation of ascorbic acid following prolonged stress will increase plasma concentrations, but prolonged supplementation should be avoided.

## 1. Introduction

There is very little information currently available about ascorbic acid (AA) supplementation in horses. Most domestic animals, including horses, have the ability to synthesize vitamin C from D-glucose or D-galactose via the glucoronic pathway [1,2]. However, the limited AA stores in the adrenal gland are mobilized by prolonged stressors such as transportation in horses and hepatic synthesis may be inadequate to maintain adequate plasma concentrations [3]. Supplementation of AA orally during training and following stress or illness in racehorses [4] and in competitive endurance horses [5] has proven to be an effective method for increasing plasma AA concentration. However, it has also been documented to be ineffective in increasing plasma AA concentrations in unstressed horses [6]. In a preliminary study in this laboratory, prolonged supplementation (10 days) of AA to weanling horses that had been transported for 36 hours resulted in significant suppression of plasma AA for over 4 weeks after supplementation was stopped [7].

Transportation in a horse trailer evokes stress responses in horses indicated by increased heart rates, increased plasma cortisol concentrations, and decreased plasma AA [3,8,9,10]. The physiologic responses are present throughout the duration of transport even if the horses are not exhibiting obvious behavioral signs of distress [10]. Most indices of stress return to baseline values following a post-transport recovery period of 24 hrs [10], however, Laegreid *et al.* [11] observed increased plasma cortisol concentrations in horses for up to a one-week period following 36 hours of transport. The degree of stress generated by transport is apparently greater in young, inexperienced horses than in older, experienced animals [12,13].

An important result of stress is an increased demand for ascorbic acid [14]. Both physical and behavioral stressors cause increased secretion of adrenocorticotrophic hormone (ACTH) and glucocorticoids such as cortisol [15]. Increased plasma cortisol causes release of AA from the adrenal gland of mammals [16,17] and depletion of body stores, and is associated with a reduction in immune competence [2,11,18]. It has been hypothesized that supplementation with AA might reduce the incidence of stress related diseases following transport such as “shipping fever” in horses [7,19]. Shipping fever is a common term for pleuropneumonia, a bacterial infection of the lungs causing nasal discharge, coughing, elevated rectal temperatures and inappetance [19]. A major risk factor for the development of pleuropneumonia is transportation greater than 500 miles [20,21]. Supplementation of AA to aged horses that had pituitary dysfunction and high plasma cortisol with depressed plasma vitamin C concentrations improved antibody response to vaccines [22].

In preliminary studies conducted in 1999 and 2000 [7] weanlings were supplemented with AA for 5 days (1999) and 10 days (2000) following prolonged (>36 hrs) transportation stress. Results suggested that supplementing horses with vitamin C for 5 days may be beneficial in reduction of the incidence of “shipping fever”. In the first study, 4/5 unsupplemented weanlings had elevated rectal temperatures, nasal discharge, coughs and inappetance 1 to 2 weeks after arrival whereas only 1/5 of the weanlings supplemented with 5.0 g AA twice a day orally for 5 days after arrival had similar clinical signs. In that study none of the horses had any handling other than processing through chutes for vaccines and anthelminthic administration. The weanlings were highly resistant to being handled in the first week after arrival and most were stressed for the first 5 days. In the second preliminary study (2000) [7], none of the horses (n = 12) developed clinical signs of illness. That group had received 5 days of pre-transport handling by the authors and their students and only foals that calmly accepted handling were acquired for the study. The weanlings that year were noticeably calmer upon arrival and quickly accepted the routine. Following cessation of 10 days supplementation of 5.0 g AA twice daily, the supplemented horses had decreased (*P* < 0.05) plasma AA for 3 weeks relative to unsupplemented horses. Vaccines given 14 days after arrival in 2000 caused lower titer responses (*P* < 0.05) in the previously supplemented horses than in the controls. A decrease in plasma AA following cessation of oral supplementation of AA after 8 days has been reported [4], though the statistical analyses of the decrease was not provided in the results. In humans prolonged AA supplementation reportedly increases the rate of urinary excretion [19]. There is also the possibility that hepatic synthesis of AA may be down-regulated with prolonged exogenous supplementation in species such as the horse where this occurs, but it has not been reported previously, to the authors’ knowledge. 

The hypotheses for the current study therefore were (1) supplementation of AA for 5 days following prolonged transport (>36 h) would increase plasma AA and response to vaccination in naïve weanling horses relative to naïve weanling horses that did not receive AA supplementation, but were otherwise handled in the same fashion, (2) prolonged (10 day) supplementation of AA to weanlings under the same conditions would result in significant and prolonged decreases in plasma AA concentrations following cessation of the supplementation, and (3) supplementation of AA to weanlings that had low plasma AA would improve immune response to vaccines. 

## 2. Materials and Methods

### 2.1. Animals and Experimental Design

This protocol was approved by the Rutgers University Institutional Animal Care and Use Committee. In August 2005, 12 draft-cross foals (6 males and 6 females) were selected from ranches in North Dakota (ND, n = 6) and Canada (n = 6). The foals were out of Belgian, Percheron or half-draft mares and were sired by American Quarter Horse (AQHA), American Paint (APHA), Thoroughbred or Hanoverian stallions. All were born between 1 April and 30 May 2005. None of the foals had received any training prior to the selection process and were running free in large pastures in a semi-feral state. Only foals that allowed the investigators to approach and handle them were selected. After selection the only handling the young horses received was to be processed through chutes where they received injections of vaccines (North Dakota only), oral anthelminthic paste and had blood drawn for Coggins tests. No vaccines were administered in Canada but the North Dakota horses received vaccines for Eastern Equine Encephalitis (EEE), Western Equine Encephalitis (WEE), West Nile Virus (WNV), Rhinopneumonitis and Influenza (EHV-1) that were administered all at the same time when they were weaned as a single group from their dams. 

Two weeks after weaning the horses were transported (Canada = 1,830 miles, 56 hours, ND = 1,730 miles, 50 hours) to Rutgers University, New Brunswick, NJ, in a single, compartmentalized commercial stock trailer pulled by a semi-truck. The Canadian weanlings were loaded into the trailer’s front compartment then transported 5 hours to North Dakota where the other 6 weanlings were loaded into the back compartment. The young horses did not have halters on and were loose in the compartments. The horses had free access to alfalfa hay fed on the trailer floor and water in buckets hung from the sides throughout the transport. The trailer made rest stops every 3 to 4 hours during which the hay and water were replenished as necessary. Upon arrival, the weanlings were fed alfalfa/grass mix hay and a commercial feed (Nutrena Youth®; Cargill, Mankato, MN, USA). The hay was fed ad libitum and the concentrate offered in amounts calculated to provide 40% of the National Research Council [23] digestible energy recommendations for moderate growth (1 to 2 kgs/day in 2 feedings). The concentrate contained no added AA but did provide 6,000 iu retinyl palmitate/kg. The vitamin content of the hay was not analyzed but was assumed to be minimal. The weanlings were weighed for the first time a week after arrival and weighed between 185 and 260 Kg with an average of 218 kg, the weanlings gained an average of 1.3 ± 0.12 kg/day throughout the study period. 

Upon arrival the weanlings were assigned to one of three treatment groups (n = 4 each). The groups were balanced for herd of origin (2 each from ND and Canada), size, temperament (calm and friendly *versus* scared and stressed), sex, and breeding (Belgian/AQHA or APHA crosses *versus* Percheron/Thoroughbred/Hanoverian crosses). Group 0 was dosed orally with 60 cc. apple sauce only (plain, unsupplemented, grocery store variety) twice a day for 10 days. Group 1 received 5 g AA (Nature’s Bounty ® Pure Vitamin C-1,000 mg), the tablets were ground to a powder and mixed with 60 cc applesauce twice daily for 5 days after which they were given 40 cc applesauce for 5 days. Group 2 received the AA supplementation in applesauce twice daily for 10 days. The treatments were administered by oral dose syringe and were well accepted by all but one of the horses. Supplementation was initiated within 6 h of arrival, after the weanling had had halters put on (a fairly stressful event for some of them). The dose was based on human recommendations (0.1–0.2 mg/kg bodyweight) and had been used in previous studies [7] in weanlings.

Forty-five hours following their arrival, blood was drawn from the weanlings via jugular venipuncture into heparinized tubes (Vacutainer) for plasma AA analysis. Blood was not drawn upon immediate arrival because the weanlings were previously unhandled and at least two days of training were needed before venipuncture could be performed safely. Sampling then took place on a weekly basis for 6 weeks with increased sampling (every 2–3 days) for the first 2 weeks. The collections occurred twice daily (0830 h and 1630 h) on the first day of sampling and at one of two times (0830 h or 1630 h) on each subsequent collection day before administering the AA treatments. Plasma AA reportedly does not exhibit diurnal variation [2]. The tubes were placed immediately on ice and transported to the laboratory within 30 min of collection. 

### 2.2. Ascorbic Acid Analysis

After blood collection, the samples were centrifuged and1.5 mL plasma aliquots were deproteinized with 6 mL metaphosphoric acid 25 within 1 hour of collection in all but the first two collections where deproteinization was delayed by 3 hours. Aliquots of deproteinized plasma were frozen at −20 °C pending AA analysis. Plasma AA concentrations were determined by a colorimetric assay [24]. Standards of 0, 0.025, 0.05, 0.1, 0.3, 0.8, and 1.2 mg AA/100 mL were used and the resultant standard curves had R^2^ of >0.99. The samples were run in triplicate. Intra-assay coefficient of variation was 0.05, inter-assay variation was 0.1. 

### 2.3. Immunization and Titers

Booster vaccinations for Eastern Equine Encephalitis (EEE), Western Equine Encephalitis (WEE), West Nile Virus (WNV), Rhinopneumonitis and Influenza (EHV-1) were administered on day 42 (10/27/05) and day 56 (11/18/05) following arrival. The weanlings from North Dakota had been previously vaccinated for the same diseases in August, 2006 (n = 6), whereas the weanlings from Canada (n = 6) had not been vaccinated previously. The Canadian dams, however, had received the full series of vaccines just prior to foaling. Blood was drawn by venipuncture immediately before vaccinations were administered and two weeks after the last vaccines were given on day 70 (12/12/05). Antibody titers for EEE and WEE were determined by a heme-agglutination test validated by serum neutralization at the Division of Animal Health Laboratories (Dr. Robert Eisner, New Jersey Department of Agriculture, Trenton, NJ, USA).

### 2.4. Statistics

Data were analyzed using Analysis of Variance and Pearson’s Correlation coefficients, (Statistix 8.0 Analytical Software, 2003; Tallahassee, FL, USA), factoring the effects of treatment, time since arrival, herd of origin, and individual. Student T-tests were used where appropriate (*i.e.*, comparing titer responses between previously vaccinated and unvaccinated horses). Significance was set at *P* < 0.05.

## 3. Results

**Figure 1:**
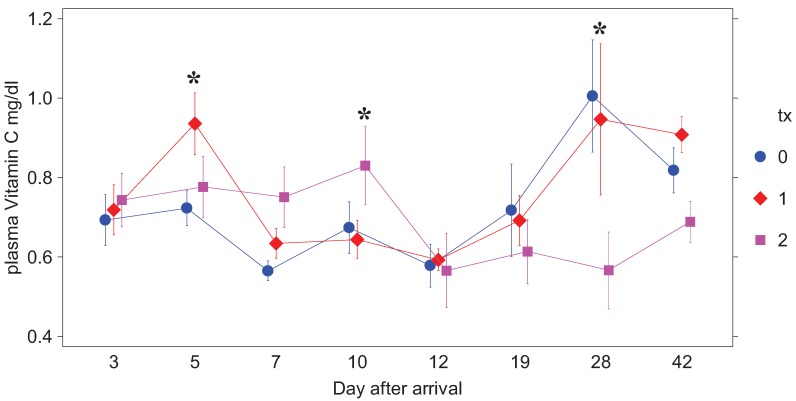
Plasma AA (mg/dL) by treatment group after arrival. Treatment 0 received only the carrier (unsupplemented applesauce), Treatment 1 supplementation of 5 g AA twice a day was stopped on day 5 post arrival and in Treatment 2 of 5 g AA twice a day was discontinued on day 10 after arrival. AA values are means ± SE.

Due to technical errors, plasma AA data for day 3 and week 5 are not available. By day 5 all but one of the horses were calm and showing no behavioral signs of stress other than resistance to venipuncture. Plasma AA results are presented in Table 1 and Figure 1. None of the weanlings had clinical signs of disease or elevated rectal temperatures during the study. At day 5 there was a only a numerical trend for horses in treatment 1 to have a higher plasma AA (*P* < 0.12) than the other two groups, which then decreased (*P* < 0.05) two days after supplementation in that group was stopped (day 7). On day 10 there was a numerical trend for plasma AA to be higher (*P* < 0.12) in treatment 2 horses, which had just received their last dose of supplement, than in the other two groups. Two days later, plasma AA was decreased (*P* < 0.05) in the treatment 2 group relative to the other two groups and remained lower (*P* < 0.05) relative to the others from day 19 through day 42 (*P* < 0.005). 

There were no differences (*P* > 0.2) among treatment groups with respect to responses to the vaccines, though the vaccine titers only had increased (*P* < 0.05) on day 70 overall relative to the two previous samples and the increases within treatment groups were not significant (Table 2). However, the previously vaccinated ND weanlings (n = 6) had a higher antibody response to WEE and EEE (*P* < 0.02) on days 56 and 70 after arrival than the unvaccinated Canada weanlings (n = 6) and were the only ones to have the significant increases in titers after the booster was given (Table 3).

**Table 1:** Mean plasma AA concentrations relative to tx group and day after arrival. Lowest Significant Difference All-Pairwise Comparisons Tests were used to determine significance across treatment groups within a day and among days of treatment.

Day	Plasma AA ± SE (mg/dL)		
	Tx 0	Tx 1	Tx 2
5	0.72 ± 0.04 ^a, wxy^	0.94 ± 0.08 ^a, x^	0.78 ± 0.08 ^a, wx^
7	0.57 ± 0.03 ^b, wx^	0.63 ± 0.04 ^ab, w^	0.75 ± 0.08 ^a, wx^
10	0.67 ± 0.06 ^a, wxy^	0.64 ± 0.04 ^a, w^	0.83 ± 0.10 ^a, x^
12	0.58 ± 0.05^ a, wx^	0.59 ± 0.03 ^a, w^	0.57 ± 0.09^ a, w^
19	0.72 ± 0.12 ^a, wxy^	0.69 ± 0.06 ^a, wx^	0.61 ± 0.08 ^b, wx^
28	1.01 ± 0.14 ^a, yz^	0.95 ± 0.19 ^a, x^	0.57 ± 0.10 ^b, w^
42	0.82 ± 0.06 ^ab, yz^	0.90 ± 0.04 ^a, x^	0.69 ± 0.05^ b, wx^

^a,b,c,d^ are used to denote a difference (*P* < 0.05) between mean AA values within days across treatments (rows).

^w,x,y,z^ are used to denote a difference (*P* < 0.05) across days within a column.

* Treatments differ at that time point, (*P* < 0.05)

**Table 2:** Eastern Equine Encephalomyelitis (EEE) titers of weanlings before and after vaccination by treatment group and day after arrival. There were equal numbers (n = 2 each) of North Dakota and Canadian horses in each group. Vaccines were administered on day 46 and boosters were given on day 56. Vaccines were not given on day 70. Treatment 0 received only the carrier (unsupplemented applesauce), Treatment 1 supplementation of 5 g ascorbic acid twice a day was stopped on day 5 post arrival and Treatment 2 of 5 g ascorbic acid twice a day was discontinued on day 10 after arrival. Values are means ± SE. There were no differences (*P* > 0.2) either among treatments or over time within treatment groups.

Treatment	Day 42	Day 56	Day 70
0	2 ± 2	7 ± 5	17 ± 8
1	0 ± 0	2 ± 2	7 ± 7
2	5 ± 5	5 ± 5	8 ± 4

**Table 3:** Eastern Equine Encephalomyelitis (EEE) titers of weanlings before and after vaccination relative to origin of horse. North Dakota horses had been vaccinated two months previously but their dams had not received pre-natal vaccines. Canadian horses had not been previously vaccinated but their dams had received prenatal vaccinations. Values are means ± SE.

Origin	Day 42	Day 56	Day 70
North Dakota	0 ± 0 ^a, y^	10 ± 4 ^a, z^	22 ± 5 ^b, z^
Canada	5 ± 3 ^a, y^	0 ± 0 ^a, y^	2 ± 2 ^a, y^

^a,b^ Means with different superscripts differ (*P* < 0.05) over time (Rows);

^y,z^ Means with different superscripts differ (*P* < 0.05) between Origins (Columns).

## 4. Discussion

The plasma AA concentrations reported in this study are somewhat higher than those reported in some other studies of normal, exercised and stressed horses [4,5,25,26] where values varied between 0.2 to 0.8 mg/dL (5 to 40 µmol/L). Plasma AA concentrations in the current study varied between 0.5 to 1.2 mg/dL, as reported by Jaeschke *et al.* [2] and previous studies by the author [3,7]. This may have been due to differences in analytic techniques, since high performance liquid chromatography was used in the more recent studies *versus* the colorimetric assays used by the authors. However the ranges did overlap and the relatively low inter-assay coefficient of variation gives confidence in the validity of the results. There were no differences (*P* > 0.3) in mean plasma AA concentrations in this study when compared to data obtained previously from weanlings from the same ranch in North Dakota and handled under the same conditions [7]. 

Ascorbic acid supplementation tended to increase plasma AA concentrations relative to unsupplemented weanlings following prolonged transport, though the increases were not statistically significant. There have been similar observations of response to supplementation in horses affected with recurrent airway obstruction [26], in race training [4] and during and after a 140 km endurance race [25]. Loscher *et al.* [6] stated that plasma AA concentration showed no significant increase when administered orally to unstressed horses, and it is presumed that AA in excess of need is excreted in the urine [1]. On the other hand, Snow *et al.* [4] found that repeated oral administration of AA to horses in strenuous training (stress condition) resulted in significant increases in plasma AA. The lack of clear significance in this study could have been due to variability in the weanlings’ responses to handling and training, which due to the relatively small sample size (n = 4 for each tx) may have obscured differences Supplementation of AA before transportation would be of no benefit since it would presumably be merely excreted. Short term stressors (<12 hours) can actually cause an increase in plasma AA as it is released from body stores in the adrenal gland (and ovaries in females) [3,25]. However prolonged stresses do decrease plasma AA [3,4,5,6,7,25,26] and supplementation of AA to horses undergoing severe stress (transportation, competition, chronic illness) for over 12 hours may benefit from supplementation. 

The horses had been weaned two weeks prior to transportation, which is a stressor that can cause two-fold increases in plasma cortisol concentrations for up to four weeks in young horses after separation from their dams [27,28]. It has also been shown that there is a correlation between stress and low plasma AA concentrations in young horses following weaning [29]. Therefore the weanlings potentially were experiencing post-weaning stress in addition to transportation stress in the first two weeks after arrival. However, though decreased appetite and increased reactivity to noises and handling was noted upon arrival in all of the weanlings, these behavioral indications of psychological stress had subsided after 5 days in all but one of the horses.

Supplementation of AA did not affect immune response to vaccines administered 6 weeks after transport of the weanlings. The delay in administering the vaccines was beyond our control and probablyhave contributed to the lack of effect. When the booster vaccinations were given, the treatment groups all had plasma AA concentrations within the normal ranges seen in previous studies and did not differ between treatment groups (data not shown). There were differences in antibody response between weanlings that had been previously vaccinated and those that had not. The increased titers observed in Canadian horses for the pre-vaccine sample could have been due to the presence of maternal antibodies that also could have interfered with subsequent vaccine responses [30].

The average half-life of AA is thought to be between 16 and 20 days [31] and the water-soluble properties of AA cause urinary excretion of the vitamin [18,31]. The prolonged decrease in plasma AA concentration could have been due to reduced endogenous synthesis and/or enhanced excretion of AA. Previous studies performed on humans have suggested that there is enhanced excretion of AA following increased vitamin C supplementation [19]. However, the possibility of reduced endogenous synthesis in horses following prolonged supplementation has not been reported and should be investigated further. Additional studies are required to assess the effect of prolonged AA supplementation and the effect of cessation of supplementation on the endogenous synthesis and excretion of AA. It would be of interest to evaluate urinary excretion of dehydroascorbate, the metabolite of AA, and to do labeling studies with C-14 labeled glucose to evaluate synthesis rates before, during and after supplementation of AA to both normal and stressed horses. 

## 5. Conclusions

Based on the results of this and previous studies, it would appear that AA supplementation for 5 days following prolonged transportation in weanling horses may be appropriate. However, prolonged supplementation of AA after adaptation to a new environment is not recommended.
